# Transcriptional Regulation of Inflammasomes

**DOI:** 10.3390/ijms21218087

**Published:** 2020-10-29

**Authors:** Maxence Cornut, Emilie Bourdonnay, Thomas Henry

**Affiliations:** Centre International de Recherche en Infectiologie (CIRI), Univ Lyon, Inserm U1111, Université Claude Bernard Lyon 1, CNRS, UMR5308, ENS de Lyon, F-69007 Lyon, France; maxence.cornut@inserm.fr (M.C.); emilie.bourdonnay@inserm.fr (E.B.)

**Keywords:** inflammasome, NF-κB, IRF, NLRP3, caspase-1, epigenetic modification, transcription factor, chromatin, promoter, enhancer

## Abstract

Inflammasomes are multimolecular complexes with potent inflammatory activity. As such, their activity is tightly regulated at the transcriptional and post-transcriptional levels. In this review, we present the transcriptional regulation of inflammasome genes from sensors (e.g., NLRP3) to substrates (e.g., IL-1β). Lineage-determining transcription factors shape inflammasome responses in different cell types with profound consequences on the responsiveness to inflammasome-activating stimuli. Pro-inflammatory signals (sterile or microbial) have a key transcriptional impact on inflammasome genes, which is largely mediated by NF-κB and that translates into higher antimicrobial immune responses. Furthermore, diverse intrinsic (e.g., circadian clock, metabolites) or extrinsic (e.g., xenobiotics) signals are integrated by signal-dependent transcription factors and chromatin structure changes to modulate transcriptionally inflammasome responses. Finally, anti-inflammatory signals (e.g., IL-10) counterbalance inflammasome genes induction to limit deleterious inflammation. Transcriptional regulations thus appear as the first line of inflammasome regulation to raise the defense level in front of stress and infections but also to limit excessive or chronic inflammation.

## 1. Introduction

A common theme in inflammatory signaling pathways is their tight control. The timely detection of pathogens and an appropriate magnitude of response are often dependent on the upregulation of specific sensors and/or their downstream adaptors/effectors. Yet, maintaining high expression of pattern recognition receptors or inflammatory mediators, in addition to being energetically costly, might trigger detection of endogenous molecules and spontaneous inflammation, causing autoinflammatory syndromes [1].

Two main categories of regulation act synergistically and in an intertwined manner to ensure appropriate responses; the first one at the transcriptional level and the second one at the post-transcriptional level (which includes post-transcriptional sensus-stricto, translational, and post-translational levels). In this review, we will focus on the first line of inflammasomes regulation: the transcriptional regulation. Post-transcriptional regulations of inflammasome components have been reviewed recently [2,3,4,5,6]. The roles of noncoding RNAs (which include microRNAs (miRNA) and long noncoding RNAs (lncRNA)) will not be described since miRNAs affect mRNA stability and translation rate downstream of transcription and since inflammasome regulation by lncRNA is either indirect [7] or mediated by a direct action onto inflammasome proteins [8].

After a brief introduction on inflammasomes and transcription regulation, we will focus on the transcriptional regulation of specific inflammasome genes illustrating whenever possible both the mechanisms and the functional consequences. Inflammasome regulation differs between humans and mice; we will thus present the regulations occurring in these two species. Of note, all uppercase letters are used for human *GENES* symbols and capitalized words for murine *Genes* symbols.

## 2. Overview of Inflammasome Complexes

**Figure 1:**
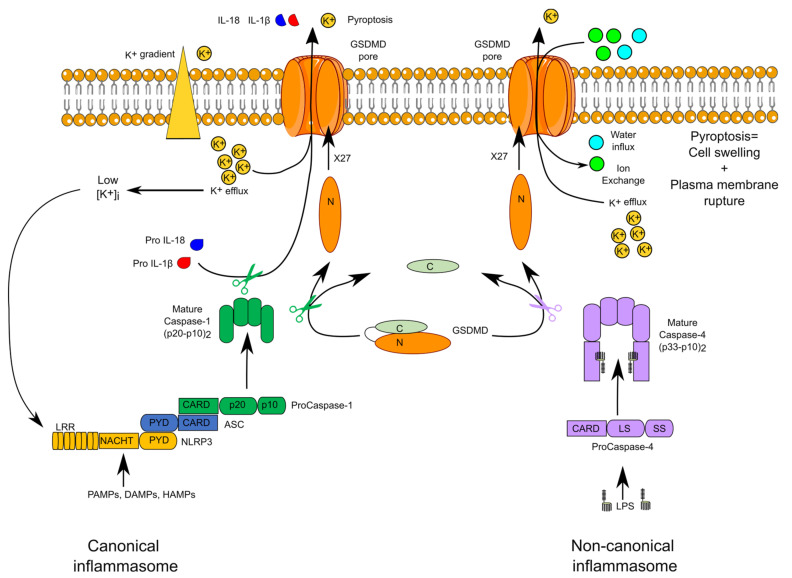
Canonical and noncanonical inflammasomes. The schematic structure of the canonical NLRP3 and noncanonical caspase-4 inflammasomes are displayed. Mature self-cleaved caspase-1 and caspase-4 cleave gasdermin D (GSDMD) leading to oligomerization of the N-terminal fragment in the membrane. The GSDMD pore promotes IL-1β and IL-18 secretion and K^+^ efflux. The latter activates the NLRP3 inflammasome.

Inflammasomes are multimolecular protein complexes assembled in the cytosol in response to pathogen-associated molecular patterns (PAMPs), damage-associated molecular signals (DAMPs), or homeostasis-altering molecular processes (HAMPs) [9] (Figure 1). Formation of these complexes leads to the activation of inflammatory caspases. Canonical inflammasomes and noncanonical inflammasomes activate caspase-1 and caspase-4/caspase-5 in humans, whereas they activate caspase-1 and caspase-11 in mice. The adaptor ASC (Apoptosis-associated speck-like protein containing a CARD) is the central piece of canonical inflammasomes and connects inflammasome sensors with caspase-1. Inflammasome sensors include members of the NLR (nucleotide-binding domain and leucine-rich repeat containing) family (NLRP1, NLRP3, NAIP, and its adaptor NLRC4), AIM2 (Absent in Melanoma 2) and pyrin. The nature of the sensor defines the name of the inflammasome complex (e.g., the NLRP3 inflammasome).

In noncanonical inflammasomes, caspase-4, or its murine homologue caspase-11, acts directly both as sensor of cytosolic lipopolysaccharide (LPS) and as the effector caspase [10]. The physiological role of caspase-5 remains unclear although caspase-5 senses cytosolic LPS and *Pseudomonas aeruginosa* (*P. aeruginosa*) outer membrane vesicles [11].

Upon activation within inflammasomes, caspase-1 triggers two main events: a fast inflammatory cell death, termed pyroptosis, and the cleavage of the proform of two cytokines (IL-1β and IL-18), resulting in the secretion of their active forms. Pyroptosis is due to the caspase-1-mediated cleavage of a single protein, termed gasdermin D (GSDMD). The cleaved N-terminal fragment of GSDMD oligomerizes into the plasma membrane to form a pore [12,13]. Structural work on GSDMA3, another GSDM family member, indicates that the mature GSDM pore is formed by 27 GSDM monomers creating a 180 Å diameter channel permitting the transport of IL-1 cytokines and of numerous ions, leading in most cases to an osmotic cell death [14]. In noncanonical inflammasomes, caspase-4 and caspase-11 cleave GSDMD and thus directly trigger pyroptosis [15]. The GSDMD pore is a nonselective pore mediating the exchange of numerous ions and molecules. Efflux of K^+^ is particularly important since it activates the NLRP3 inflammasome downstream of noncanonical inflammasomes [16,17].

Inflammasomes are implicated in the antimicrobial responses to numerous pathogens including *Salmonella enterica*, *Legionella pneumophila*, *Francisella tularensis*, and vaccinia virus [18]. Yet, inflammasome activation can also be deleterious as demonstrated in murine models of septic shock or Alzheimer’s disease [19] and in patients suffering from autoinflammatory syndromes due to gain of function mutations in inflammasome sensor genes [20]. Inflammasomes are thus tightly regulated with numerous positive and negative regulations ensuring, in most situations, a proper balance between immune defenses against infection and the absence of chronic sterile inflammation.

Here, we review the first level of inflammasome regulation, namely the transcriptional regulation. Importantly, transcriptional regulation affects most, if not all, inflammasome molecules from sensors to downstream inflammasome products (pro-inflammatory cytokines, GSDMD) and, has a profound impact on the inflammasome responses [21,22,23,24,25,26]. Before developing the specific transcriptional regulation of inflammasome genes, we will give a brief overview of transcription regulation.

## 3. Overview of Transcription Regulation

Transcription is the generation of an RNA molecule from a DNA template. Protein-encoding genes are transcribed by the RNA polymerase II (Pol II), whose activity is controlled at the recruitment phase, at the initiation of elongation (that requires to overcome Pol II pausing), and at its processivity level.

One key aspect of transcriptional regulation is mediated by regulating Pol II and transcription factors (TFs) access to DNA. DNA accessibility is highly controlled and limited in most of the genome due to high chromatin compaction. Nucleosomes (i.e., a ≈150 bp-long DNA piece wrapped around a histone octamer) represent the first level of chromatin organization. Nucleosomes can be located at the transcription start site (TSS), on the TATA box, or on specific transcription factor binding sites and can therefore block transcription. Chromatin remodeling allows TFs to access their target genes and control transcription. This process involves two families of enzymes: the ATP-dependent nucleosome remodeling complexes (e.g., from the switch/sucrose nonfermenting-SWI/SNF family) and the histone modifying enzymes (e.g., histone acetyl transferases-HAT). The histone-modifying enzymes add methyl or acetyl groups to histone tails. These covalent modifications change the affinity of histones for DNA and modify their ability to bind transcription coactivators or corepressors. As an example, histone H3 lysine 27 trimethylation-(H3K27me3) is a repressive epigenetic modification. In contrast, H3K4me3 and H3K9 acetylation-(H3K9ac) are activating modifications and H3K27ac is a mark of transcriptional activity. H3K4me1 is an enhancer-specific mark.

TFs binding sites are located in DNA sequences called promoter in the vicinity of the TSS and promoting Pol II recruitment. TFs binding sites can also be located megabases away from the TSS in regions called enhancers or silencers. These regions, often far away when considering the linear distance along the genome, can interact through DNA loops and be in close proximity when considering the three-dimensional chromatin structure. Transcription regulation thus integrates signals from both promoter and enhancer/silencer regions.

The cell type-specific and the signal-dependent transcriptional regulations are controlled by the hierarchical and coordinated binding of unique combinations of transcription factors. TFs can be classified in two categories: the pioneer TFs (e.g., PU.1) and the signal-dependent TFs (e.g., nuclear factor κB, NF-κB). Pioneer TFs bind to their specific recognition motifs, most often in enhancers, even in the context of highly compact chromatin. These pioneer TFs displace nucleosomes, open chromatin structure and promote DNA accessibility, revealing novel TFs binding sites for nonpioneer TFs. Pioneers TFs are expressed during lineage development and drive constitutive and signal-dependent, lineage-specific transcription programs. They are thus also named lineage-determining TFs. Binding of pioneer TFs on inactive enhancers transforms them into primed, poised (see *IL1B* enhancer below), or active enhancers [27] that present three different epigenetic states with specific characteristics. In contrast to inactive enhancers displaying highly compact chromatin, active enhancers present a wide nucleosome-free region, recruit Pol II, which results in strong expression of target genes. Primed enhancers display constitutive binding of lineage-determining/pioneer TFs, a nucleosome-free region of open chromatin but very low recruitment of paused Pol II. Poised enhancers are primed enhancers displaying the additional presence of repressive epigenetic marks (e.g., H3K27me3). During activation, they can rapidly change from poised to active state, a change associated with a switch from H3K27me3 to H3K27ac.

Productive transcription requires both Pol II recruitment and transition from a paused Pol II to an elongating Pol II. These two steps are indirectly controlled by TFs through histone modifications. Pol II recruitment can follow HAT recruitment and activation through direct interaction with TFs. The ensuing histone acetylation marks the chromatin for assembly of the preinitiation complex that includes Pol II bound to DNA close to the TSS. Transcription elongation is also mediated by TFs, which recruit the positive elongation factors bromodomain-containing protein 4 (Brd4) and P-TEFb (positive transcription elongation factor b). P-TEFb phosphorylates Pol II to promote productive elongation.

Transcription regulation is thus a complex process resulting from the hierarchical and collaborative actions of multiple lineage-determining TFs and signal-dependent TFs. These TFs act by modifying, in multiple stable or dynamic ways, chromatin structure to ensure Pol II recruitment and productive elongation in a timely controlled manner. Due to the high complexity of this process and the multitude of TFs binding sites affecting the transcription of a particular gene, almost each gene has its own spatiotemporal expression although common themes can be observed.

## 4. Transcriptional Regulation of Inflammasome Sensors

### 4.1. NLRP1

NLR Family Pyrin Domain Containing 1 (NLRP1) is an inflammasome sensor sensing cytosolic proteolytic activity. Anthrax lethal factor, a toxin with endoprotease activity, and IpaH7.8, a *Shigella flexneri* effector with ubiquitin ligase activity, trigger NLRP1B degradation and activation [28,29]. Human NLRP1, the three murine paralogues NLRP1A, B, and C, each one with different isoforms in different mouse strains, detect different stimuli [29].

In mice, *Nlrp1a* expression is restricted to the hematopoietic compartment and is expressed in hematopoietic stem cells, progenitor cells of both myeloid and lymphoid origins, and terminally differentiated cells such as macrophages [30].

**Figure 2:**
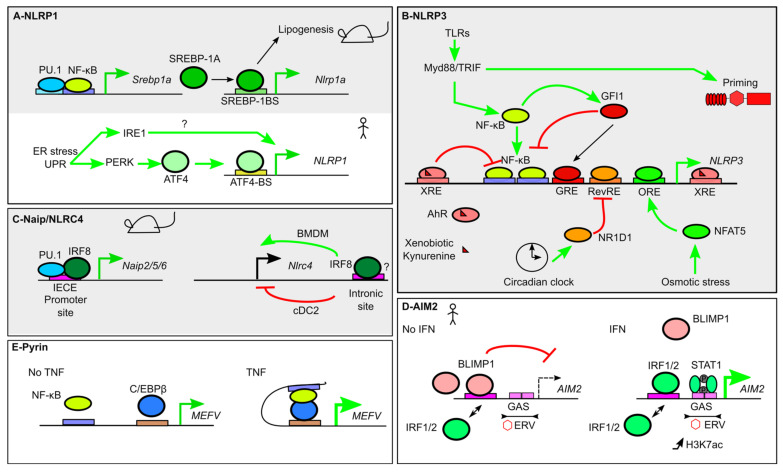
Transcriptional regulation of inflammasome sensors. (**A**) In mice (gray background), *Nlrp1a* expression is indirectly induced by Nuclear factor κB (NF-κB), which controls *Srebp1a* expression. SREBP1A couples *Nlrp1a* expression and lipogenesis. In humans, *NLRP1* expression is induced by ER stress and the Unfolded protein response (UPR) and two of its effectors, Inositol-requiring enzyme 1 (IRE1) and PKR-like ER protein kinase (PERK). PERK induces Activating Transcription Factor 4 (ATF4) expression that directly upregulates *NLRP1* expression. (**B**) *NLRP3* expression is under the control of numerous TFs. NF-κB activation downstream of Toll-like receptors (TLRs) induces NLRP3 expression, while NLRP3 inflammasome priming is independent on transcription. GFI1 negatively regulates *NLRP3* expression. *GFI1* expression is inducible by NF-κB, thus creating a negative feedback loop. Xenobiotics and anti-inflammatory metabolites promote Aryl hydrocarbon receptor (AhR) binding to Xenobiotic Response Elements (XRE) that blocks NF-κB-mediated *NLRP3* induction. Similarly, *NLRP3* promoter integrates signals from the circadian clock regulator Nuclear receptor subfamily 1 group d member 1 (NR1D1) and from Nuclear factor of activated T cells 5 (NFAT5), which binds to an Osmotic Response Element (ORE), following osmotic stress. (**C**) Several *Naip* are regulated at steady state by Interferon Regulatory Factor 8 (IRF8), which binds Ets-Interferon Regulatory Factors (IRF) composite Element (EICE), together with PU.1. Binding of IRF8 in the intronic region of *Nlrc4* controls positively or negatively *Nlrc4* expression in bone marrow-derived macrophages (BMDM) and type 2 conventional dendritic cells (cDC2), respectively. (**D**) At steady state (left panel) *AIM2* expression is repressed due to B lymphocyte-induced maturation protein-1 (BLIMP1) binding to an IRF/BLIMP1 composite site. In the presence of IFN-γ, IRF1/2 displace BLIMP1. Furthermore, Signal Transducer and Activator of Transcription 1 (STAT1) binding to *GAS* triggers *AIM2* expression, which correlates with a local increase in H3K7 acetylation (H3K7ac). The tandem *GAS* sites are present in an endogenous retrovirus sequence (ERV). (**E**) Pyrin encoded by the *MEFV* gene is under the regulation of NF-κB. In the presence of Tumor necrosis factor (TNF), a chromatin loop favors NF-κB and CCAAT/enhancer binding protein β (C/EBPβ) interaction to upregulate *MEFV* expression.

Expression of *Nlrp1a* and *Nlrp1c* is positively regulated by SREBP-1A (Sterol regulatory element binding protein-1a), a basic helix–loop–helix leucine zipper (bHLH-LZ) TF [31]. SREBP-1A binds directly onto a canonical SREBP-1 binding site in the *Nlrp1a* proximal promoter [31]. *Srebp-1a* expression is itself under the direct control of NF-κB, in synergy with the monocyte/macrophage specific TF PU.1 (an Erythroblast Transformation Specific (ETS) family TF). NF-κB thus indirectly controls *Nlrp1a* expression in macrophages (Figure 2A) [31]. SREBP-1A also controls LPS-triggered lipogenesis in macrophages, an anabolic pathway required for optimal inflammasome responses. SREBP-1A regulation thus directly couples lipogenesis and control of the NLRP1A inflammasome [31]. SREBP TFs being key signaling nodes responding to metabolic clues, the control of *Nlrp1a* expression might thus participate to the control of metabolic inflammation. Interestingly, mice lacking the three murine *Nlrp1* alleles (or *Il18*) develop metabolic syndrome and spontaneous obesity strengthening the functional link between metabolism and *Nlrp1* sensors [32].

In humans, *NLRP1* is broadly expressed but at particular high levels in keratinocytes, a feature that is not observed in mice [33]. The molecular basis of the differential expression of *NLRP1* between humans and mice remains unknown. It is controlled at the transcriptional level since inflammasome transcripts are very low in murine keratinocytes [34].

Endoplasmic reticulum (ER) stress strongly induces *NLRP1* expression in THP-1 monocytes and in various human cell lines. This induction is dependent on two effectors of the unfolded protein response (UPR): PKR-like ER protein kinase (PERK) and Inositol-requiring enzyme 1 (IRE1). Although the mechanisms downstream of IRE1 remain unsolved, PERK activation leads to Activating Transcription Factor 4 (ATF4) expression. ATF4 is a TF from the ATF/cAMP Response Element-binding protein (CREB) family. ATF4 binds to the promoter of human *NLRP1* to induce its expression (Figure 2A) [35]. It is thus tempting to speculate that ER stress could prime the NLRP1 inflammasome to increase its ability to detect HAMPs and PAMPs.

### 4.2. NLRP3

NLRP3 is an inflammasome sensor detecting multiple cellular homeostasis perturbations such as membrane damage, mitochondrial defects, or perturbations of ionic concentrations. NLRP3 inflammasome activation is a two-step process and requires a signal 1 (priming signal, e.g., LPS) and a signal 2 (activation signal, e.g., nigericin). Priming is triggered by pro-inflammatory signals such as Toll-like receptor 4 (TLR4) engagement and has long been associated with transcriptional upregulation of *NLRP3* [36,37]. It is now clear that NLRP3 priming is mediated by post-translational modifications independent of transcription (Figure 2B) [38,39,40]. Yet, *Nlrp3* upregulation accelerates the kinetics and the level of caspase-1 activation following signal 2 addition [38]. The importance of this upregulation is likely enhanced by the low level of expression of *Nlrp3* at steady state.

*NLRP3* upregulation occurs within 2 h of LPS addition but can also be triggered by other TLR ligands, by the nucleotide-binding oligomerization domain 2 (Nod2) ligand (muramyl dipeptide) or by pro-inflammatory cytokines (Tumor necrosis factor (TNF), Interleukin (IL)-1α, IL-1β) in the absence of PAMPs [36,37]. This upregulation is dependent on NF-κB as first demonstrated using the BAY 11-7082 inhibitor. Two NF-κB binding sites are present in the *NLRP3* promoter. Upon LPS treatment, the NF-κB subunit RelA/p65 binds the *NLRP3* promoter. LPS-induced *NLRP3* upregulation is lost upon mutation of these two binding sites indicating that these two sites control in a redundant manner the increase in *NLRP3* promoter activity [41]. Upregulation of *Nlrp3* (and *Il1b*) following NF-κB activation is partially dependent on an atypical IκBs (inhibitor of κB), a coactivator of NF-κB in bone marrow-derived macrophages (BMDMs) [42]. IκBζ binds to another NF-κB subunit, p50, in the *Nlrp3* promoter. The NF-κB heterodimer RelA/p50 may thus be responsible for LPS-induced *Nlrp3* expression. IκBζ recruitment increases H3K4me3, a mark of active transcription [43] suggesting that this coactivator promotes *Nlrp3* induction through epigenetic modifications. Interestingly, the parasite *Leishmania amazonensis* subverts this process by targeting the epigenetic control of NF-κB-related pro-inflammatory genes. During infection, the promoters of these genes display hypoacetylation of the histone H3K9/14 and hypotrimethylation of histone H3K4. These inhibitory epigenetic modifications dampen *Nlrp3* (and also *Aim2*, *Nlrc4*, *Pycard, Il1b*, and *Il18*) expression and promotes survival of the parasite within the host [44].

In addition to its control by pro-inflammatory signals, *Nlrp3* expression is controlled by the circadian clock and presents a peak of expression during the night [45,46]. The circadian rhythm in *Nlrp3* transcript level is dependent on a master regulator of the clock, the transcription factor NR1D1 (nuclear receptor subfamily 1 group D member 1, also known as Rev-erbα). Circadian oscillations of *Nlrp3* and *Nr1d1* expressions are in antiphase. Accordingly, NR1D1 binds directly to a Rev-response element (RevRE) in the *Nlrp3* promoter to repress its expression (Figure 2B). Interestingly, an agonist of NR1D1 attenuates both dextran sodium sulfate (DSS)-induced colitis and D-galactosamine-induced fulminant hepatitis suggesting that NR1D1-mediated circadian control of *Nlrp3* expression controls inflammation in vivo [45,46].

Besides NF-κB and NR1D1, direct binding of several TFs on the *NLRP3* promoter have been reported with either negative or positive impact on *NLRP3* expression. Downregulation of *NLRP3* expression is controlled by the aryl hydrocarbon receptor (AhR) [47] and by B cell lymphoma 6 (BCL6), a transcriptional repressor that antagonizes NF-κB-mediated gene transcription [48]. AhR is a ligand-activated TF binding numerous environmental contaminants (e.g., dioxin) and endogenous ligands (e.g., the tryptophan derivative, kynurenine). AhR binds two XRE (Xenobiotic Responsive Element) sequences in the *Nrlp3* promoter, and its binding is increased in a ligand-dependent manner. The two XRE sites surround the two NF-κB sites in the *Nlrp3* promoter suggesting that AhR may interfere with NF-κB recruitment either directly or indirectly by modifying the local chromatin architecture. AhR and its ligands also negatively control *Il1b* expression indicating that environmental pollutants (and the anti-inflammatory tryptophan catabolites) dampen inflammasome responses (Figure 2B) [47].

Growth Factor Independence 1 (GFI1), a protein induced by NF-κB, binds the *Nlrp3* promoter to inhibit *Nlrp3* expression in a negative feedback loop [49]. In contrast, direct binding of Nuclear factor of activated T cells 5 (NFAT5—a transcription factor induced in response to high salt, hypoxia and mechanical stress) on an osmotic response element (ORE) in the promoter of *Nlrp3* positively regulates *Nlrp3* expression [50]. Overall, regulation of *Nlrp3* expression emerges as a complex network primarily driven by pro-inflammatory signals but integrating environmental and intrinsic signals.

*Nlrp3* expression has been mostly studied in macrophages and displays a cell-type-specific regulation in dendritic cells (DCs). Of note, the DC-specific gene regulation affects not only *Nlrp3* but also other inflammasome genes as detailed below. Indeed, plasmacytoid dendritic cells (pDC) express neither *Nlrp3* nor *Il1b* and are resistant to inflammasome activation [51]. Conventional DCs (cDC) have a limited ability to respond to inflammasome stimuli [21,51]. Interferon Regulatory Factors (IRFs) belong to a family of TFs, which includes 9 members in human and mice. pDCs express high levels of IRF8, while cDC1 and cDC2 express high levels of IRF8 and IRF4, respectively. IRF4 and IRF8 bind the promoter regions of *Nlrp3*, *Il1b*, and *Aim2* and intronic regions of *Pycard* and *Nlrc4*, which correlate with the low expression of these genes in cDC1 and cDC2. Haploinsufficiency in *Irf8* increases *Nlrc4*, *Pycard,* and *Il1b* expression in cDC1 and increases the inflammasome response to the NLRC4-engaging pathogen *Salmonella typhimurium* (*S. typhimurium*). In contrast, ectopic expression of *Irf8* in macrophages decreases *Nlrp3*, *Nlrc4*, and *il1b* expression, while ectopic expression of *Irf4* decreases *Nlrc4*, *Il1b*, and *Pycard* expression [21]. Importantly, the low inflammasome activity in cDC limits their pyroptosis in response to bacterial pathogens and favors antigen presentation and T cell priming.

### 4.3. NAIP and NLRC4

The Neuronal apoptosis inhibitory protein (NAIP)/NLRC4 inflammasome is atypical since NLRC4 is not a direct sensor but requires a NAIP protein to sense PAMPs. A single NAIP protein is encoded in the human genome and binds bacterial type III secretion system (T3SS) needle proteins [52] and flagellin [53]. Several NAIP paralogs are present in mice and bind T3SS needle proteins (NAIP1), T3SS inner rod proteins (NAIP2), or flagellin (NAIP5/6) [54,55].

IRF8 was identified in mice as a positive regulator of *Naip2, 5, 6*, and *Nlrc4*. IRF8 binds the promoter regions of *Naip2, 5, 6*, and an intronic region of *Nlrc4*. IRF8 has a very low intrinsic DNA-binding activity and, at steady state, binds with PU.1 at Ets-IRF composite elements (EICE) (Figure 2C) [56]. NAIP/NLRC4 inflammasomes are the major inflammasomes allowing mice to control *S. typhimurium* infection. Accordingly, *Irf8*^−/−^ mice are highly susceptible to *S. typhimurium* [25]. Interestingly, although the effects of IRF8 were demonstrated at steady state, *Irf8* is induced upon *Legionella* infection and could participate in the NAIP/NLRC4-dependent response against this flagellin-expressing pathogen [57]. Surprisingly, overexpression of *Irf8* in bone marrow-derived macrophages decreases *Nlrc4* expression. Furthermore, as presented above, in cDC1, IRF8 inhibits *Nlrc4* expression (Figure 2C) [21]. IRF8 concentration modulates its ability to cooperate with other TFs and engage different binding sites [58] possibly explaining its reported opposed role on *Nlrc4* at steady state and upon overexpression.

### 4.4. AIM2

AIM2 is a receptor of cytosolic DNA [59,60]. Since, in healthy cells, DNA is restricted to the nucleus, the presence of cytosolic DNA is either indicative of an infection or of a cellular stress associated with the loss of nuclear membrane integrity [61].

*AIM2* is an IFN-inducible gene. In murine BMDMs, *Aim2* is expressed at steady state and its expression is slightly induced by IFN, poly(dA:dT) treatment, or infections [60]. In mice, AIM2 levels are sufficient to promote its inflammasome functions, and the AIM2 inflammasome does not require a priming step [62,63]. Its role in myeloid human cells is less clear than in the murine context. Indeed, a cGAS-STING-lysosomal-NLRP3 pathway triggers cell death in response to cytosolic DNA in human myeloid cells [64], while in the presence of IFN-γ, AIM2 is involved in *T. gondii* responses. These results suggest that AIM2 functionality in human cells is variable depending on the context [65]. In contrast to murine macrophages, AIM2 levels are very low at steady state in human macrophages but are strongly induced by LPS [66] or IFN-γ [65]. The upregulation of *AIM2* transcript in inflammatory conditions is likely relevant in human diseases since *AIM2* expression level is increased in keratinocytes from psoriatic lesions compared to healthy skin [67].

In human cells, *AIM2* expression is dynamically controlled by B lymphocyte-induced maturation protein-1 (BLIMP1 also known as PR domain zinc finger protein 1-PRDM1), IRF1/2 and Signal Transducer and Activator of Transcription 1 (STAT1), a transcription factor activated by interferons [68,69]. BLIMP1 binding site overlaps with IRF1/2 binding site and these transcription factors compete to repress or activate *AIM2* expression, respectively. Indeed, *BLIMP1* knock-down increases IRF1/2 binding on the *AIM2* promoter and AIM2 expression (Figure 2D) [68]. To our knowledge, it is unknown whether BLIMP1 modulation may be involved in licensing AIM2 function in certain human cell types. In addition, two tandem Gamma-activated site (GAS) sequences are present 220 nt upstream of *AIM2* TSS. These GAS belong to the long terminal repeat (LTR) sequence of an endogenous retrovirus termed *MER41*. Treatment with IFN-γ triggers an increase in H3K27ac and STAT1 binding at the *MER41.AIM2* site. Accordingly, *MER41* sequence is required for *AIM2* upregulation in HeLa cells exposed to IFN-γ. The conservation of the *MER41.AIM2* site across anthropoid primates (but not in mice) suggests that this retrovirus sequence was co-opted for *AIM2* regulation in an ancestor of anthropoid primates [69] and illustrates how past infections have shaped the species-specific regulation of inflammasome genes. Interestingly, a *MER41* sequence displaying IRF1 and STAT1 binding in monocytes is also found in proximity of *GSDMD*, suggesting that the regulation of several inflammasome genes may result from retrovirus integration.

### 4.5. Pyrin

Pyrin is a sensor detecting Rho GTPase inhibition. Rho GTPases are altered by numerous bacterial toxins and bacterial effectors. Pyrin thus acts as a guard of this important cytoskeleton regulator [70]. Gain of function mutations in *MEFV*, the gene encoding Pyrin, cause several inflammatory syndromes, including Familial Mediterranean Fever (FMF) [71].

Pyrin is constitutively expressed in human neutrophils, monocytes, and M-CSF-differentiated monocyte-derived macrophages [72]. In monocytes, *MEFV* expression is positively regulated by IFN-α, IFN-γ, and TNF, while IL-4 and IL-10 repress it [73,74]. TNF-dependent upregulation of *MEFV* expression is due to the binding of CCAAT/enhancer binding protein β (C/EBPβ) in the *MEFV* promoter, which acts in synergy with NF-κB p65/RelA subunit. Indeed, NF-κB p65/RelA binds the *MEFV* promoter both through a canonical NF-κB binding site and indirectly through its binding to C/EBP*β* (Figure 2E) [75]. The mechanism underlying IFN-mediated upregulation is unclear although IFN-stimulated response element (ISRE) and GAS consensus sequences have been identified in silico in the *MEFV* promoter [73].

In mice, *Mefv* expression is induced by TLR ligands and inflammatory cytokines (IFN-β + TNF). TNF signaling contribution to inflammation in a murine model of FMF strongly suggests that transcriptional regulation of inflammasome sensors is important not only for the detection of pathogens but also for the prevention of deleterious inflammatory reactions [76].

## 5. Transcriptional Regulation of ASC

The transcriptional regulation of ASC and its gene *PYCARD* was evidenced upon its discovery. Indeed, *PYCARD* was identified as a gene whose expression was lost in human breast cancer cells due to methylation of a CpG island in its promoter (in normal cells, CpG islands, in contrast to sparsely distributed CpG dinucleotides, remain unmethylated). Similarly, *AIM2* was identified as a gene whose expression was lost in a melanoma cell line [77]. At the time, *PYCARD* was named “*TMS1*,” for “target of methylation-induced silencing 1” [78]. Overexpression of DNMT1 (DNA (cytosine-5)-methyltransferase) leads to aberrant hypermethylation of CpG islands in the 5′ untranslated region of *PYCARD* resulting in *PYCARD* silencing and recapitulating the transcriptional shutdown observed in numerous primary breast tumors. The pro- and antitumoral roles of inflammasomes are well established. Transcriptional silencing of inflammasome genes could thus promote tumor escape from immune surveillance.

Although *PYCARD* expression in nontumor cells is largely constitutive and insensitive to pro-inflammatory signals, invalidation of *ifi205*, a murine-specific gene from the IFN-inducible PYHIN (Pyrin and HIN domain) family leads to the loss of *Pycard* expression [79]. In addition, NF-κB p65/RelA and C/EBP*β* have putative binding sites in the *Pycard* promoter, and ectopic expression of these transcription factors in a HEK293-reporter system increases *Pycard* promoter activity. Interestingly, IFI205 interacts with C/EBP*β* and synergizes with p65/RelA to promote *Pycard* promoter activity [79]. IFI205 has no direct orthologues in humans [80]. Whether other members of the PYHIN/AIM2-like Receptor (ALR) family could regulate *PYCARD* expression in human cells remains unknown.

## 6. Transcriptional Regulation of Inflammatory Caspases

### 6.1. Caspase-1

Caspase-1 is the effector caspase of canonical inflammasomes. Its expression is largely constitutive but a loss of balance in *CASP1* expression may be relevant in pathological situations. For instance, *CASP1* expression is upregulated in the brain of multiple sclerosis (MS) patients compared to controls and may participate in MS lesions [81]. Conversely, *CASP1* is downregulated in PBMCs from septic patients, a feature that could contribute to the severe immunodepression observed in these patients [82].

*CASP1* promoter possesses an ISRE immediately upstream of its TSS participating in the control of *CASP1* expression at steady state and in the presence of IFN. Indeed, IRF2 binds *CASP1* promoter in primary human monocytes, and *IRF2*-deficient U937 monocytes present a strong decrease in *CASP1* levels at steady state (Figure 2A) [22,83]. IFN-γ, which strongly induces *IRF1*, upregulates *CASP1* expression [84]. In mice, a similar ISRE site is found in the promoter of *Casp1*, and *Irf1*^−/−^ splenocytes are deficient in *Casp1* induction following concanavalin-A stimulation [85]. In contrast, *Irf2*^−/−^ mice and murine macrophages do not present a major defect in *Casp1* level [23,86] suggesting differences in the basal regulation of *CASP1* in mice and humans.

**Figure 3:**
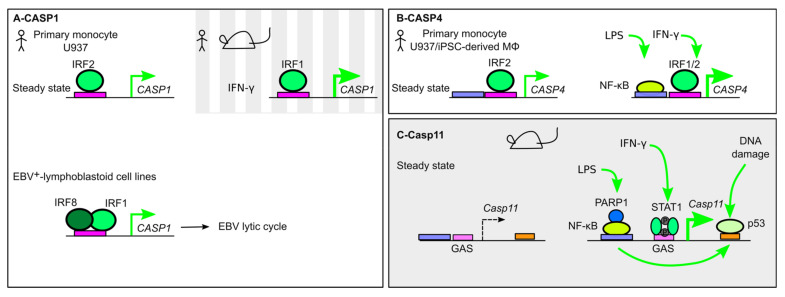
Transcriptional regulation of inflammatory caspases. (**A**) In human monocytes, *CASP1* expression is regulated at steady state by IRF2, while in the presence of IFN-γ, IRF1 regulates its expression. In Epstein–Barr virus (EBV^+^) lymphoblastoid cell lines, a ternary IRF1/8/DNA complex controls *CASP1* expression and EBV lytic cycle. (**B**) *CASP4* is constitutively expressed in human monocytes in an IRF2-dependent manner. IFN-γ and lipopolysaccharide (LPS) can upregulate *CASP4* expression by promoting NF-κB and IRF1 binding to the *CASP4* promoter. (**C**) *Casp11* expression is undetectable at steady state but demonstrates a strong upregulation in the presence of LPS or IFN-γ, which promotes the recruitment of NF-κB and STAT1. Poly [ADP-ribose] polymerase 1 (PARP1) acts as a positive cofactor of NF-κB. DNA-damage triggers p53 binding in the *Casp11* first intron and induction of its expression. p53 binding is dependent on NF-κB.

Another member of the IRFs family, IRF8, also controls *CASP1* expression as exemplified by its role during Epstein–Barr virus (EBV) infection [87]. Invalidation of *IRF8* in several EBV^+^ lymphoblastoid cell lines fully abolishes *CASP1* expression. IRF8 binds the consensus ISRE sequence in the proximal promoter of *CASP1*, and its ability to transactivate *CASP1* expression was validated using a luciferase reporter/minimal promoter assay. In this assay, IRF8 synergizes with IRF1 to control *CASP1* promoter activity suggesting that IRF8 binds DNA in a ternary complex with IRF1 [56]. Interestingly, this IRF8/CASP1 cascade facilitates EBV lytic replication, likely through the cleavage of host factors involved in the maintenance of EBV latency (Figure 3A) [87].

### 6.2. Caspase-4

Caspase-4 is an inflammatory protease, which binds intracellular LPS. Caspase-4 cleaves GSDMD, directly triggering pyroptosis and indirectly activating the canonical NLRP3 inflammasome.

IRF2 is a key regulator of CASP4 as identified through a genome-wide CRISPR-Cas9 screen performed in the premonocytic U937 cells [22]. *IRF2*^−/−^ U937 are resistant to cytosolic LPS-induced pyroptosis and ectopic expression of *CASP4* in *IRF2*^−/−^ cells fully restores, even accelerates, pyroptosis in response to cytosolic LPS delivery. The regulatory role of IRF2 on *CASP4* levels was confirmed in induced pluripotent stem cells (iPSCs)-derived macrophages at both mRNA and protein levels. IRF-2 binds *CASP4* promoter in primary human monocytes. Interestingly, in the presence of IFN-γ, IRF1 can complement the absence of IRF2 to regulate *CASP4* levels (Figure 3B). This regulation of *CASP4* by IRF2 is not observed at the protein level in the cell line EA.hy926 (an hybrid of A549 and human umbilical vein endothelial cells), suggesting that the *CASP1* expression dependency on IRFs might differ between different human cell types [23,88].

In addition to IRF1/2, the NF-κB subunit RelA upregulates *CASP4* expression via its binding on a NF-κB binding site located ≈1000 bp upstream of *CASP4* TSS [89].

### 6.3. Caspase-11

Caspase-11 is the murine homolog of human Caspase-4/5. Like caspase-4, caspase-11 directly binds intracytosolic LPS and cleaves GSDMD to trigger pyroptosis.

Naive mice present undetectable levels of *Casp11* expression in tissues. *Casp11* expression is induced in response to pro-inflammatory stimuli (e.g., LPS) or IFN [90,91]. Schauvliege et al. validated the presence of a NF-κB binding site in the *Casp11* promoter required for *Casp11* induction in response to LPS. cRel and likely other NF-κB subunits bind this site upon LPS stimulation. In the presence of IFN-γ, STAT1 binds a GAS motif in the *Casp11* promoter. Mutation of this motif invalidates both IFN-γ- and LPS-induced *Casp11* expression. Yet, since a putative NF-κB binding site overlaps with the GAS site, it is unclear whether STAT1 is required for both LPS- and IFN-γ-mediated *Casp11* induction (Figure 3C) [90].

C/EBP homologous protein (CHOP) is a TF from the C/EBP family, whose expression is induced by ER stress. CHOP is required for *Casp11* induction both in the lung of LPS-treated mice and in vitro in LPS-treated peritoneal macrophages. ER stress inducers (e.g., Thapsigargin) induce *Casp11* expression in a CHOP-dependent manner in the absence of LPS [92]. Intratracheal LPS delivery induces ER stress in the lung and *Chop*^−/−^ mice display attenuated LPS-induced lung inflammation. Although it is unclear whether CHOP acts directly on the *Casp11* promoter, CHOP may connect ER stress and the noncanonical inflammasome as described above for ATF4 and NLRP1.

Poly(ADP-ribose) polymerase 1 (PARP-1) is one of the most abundant nuclear protein. PARP-1 is a multifunctional enzyme acting at numerous levels to regulate transcription [93]. Interestingly, Yoo et al. identified that PARP-1 is necessary for LPS-induced, but not IFNγ-induced, *Casp11* expression. PARP-1 interacts with cRel and RelA and regulates NF-κB-dependent genes independently of its enzymatic activity [94]. PARP-1, as a NF-κB-co-activator, may thus regulate specifically NF-κB-mediated *Casp11* induction [95].

Interestingly, *Casp11* expression can also be induced in a p53-dependent manner following DNA damage [96]. Two p53 binding sites are present in the first intron of *Casp11* and p53 binding on *Casp11* promoter is observed by ChipSeq following gamma irradiation of thymocytes or etoposide treatment of mouse embryonic fibroblasts. NF-κB is required for p53 binding to *Casp11* promoter [96]. *CASP1* and *NLRC4* promoters display a similar p53 binding site and a similar upregulation in response to DNA-damaging agents (e.g., etoposide) [97,98], suggesting that p53 (or p53 family members) and NF-κB function cooperatively to ensure an appropriate inflammasome response to sterile and infectious stresses.

The importance of *Casp11* induction was demonstrated in murine models of septic shock. In contrast to humans, who express *CASP4* constitutively, mice are highly resistant to LPS injection (lethal dose about 40 mg/kg). Yet, if mice are primed with a low dose of LPS (400 μg/kg) or with poly(I:C), a potent IFN inducer, they become highly sensitive to very low doses of LPS (10–100 ng/kg) [99,100]. Similarly, a transgenic mouse expressing human *CASP4* under its native promoter displays a constitutive *CASP4* expression in the spleen and in the intestine and was highly susceptible to LPS injection [101]. These experiments thus suggest that induction of *Casp11* decreases the threshold of LPS detection. Although this induction is detrimental in a septic shock model, it contributes to the immune defenses against Gram-negative bacteria [24].

### 6.4. Caspase-5

Caspase-5 is an inflammatory caspase directly binding cytosolic LPS and triggering pyroptosis [10].

*CASP5* is expressed at low levels in various organs with a more predominant expression in the spleen and in the colon [102]. Its expression is highly induced in vitro following LPS treatment or IFN treatment [102,103,104]. Induction of *CASP5* is delayed in comparison to *IL1B* induction and is sensitive to the protein synthesis inhibitor cycloheximide, indicating that it is a secondary-response gene. Three putative binding sites for NF-κB are present in the *CASP5* promoter but their functionality remains to be experimentally tested [105]. The relevance of *CASP5* induction in diseases is still unclear, although a strong increase in *CASP5* level (20-fold increase) is observed in lesional psoriatic skin [105].

## 7. Transcriptional Regulation of Downstream Targets

### 7.1. GSDMD

GSDMD is the pyroptosis executioner [12,15]. N-terminal fragments generated following inflammatory caspases cleavage oligomerize in the plasma membrane to form a transmembrane pore.

**Figure 4:**
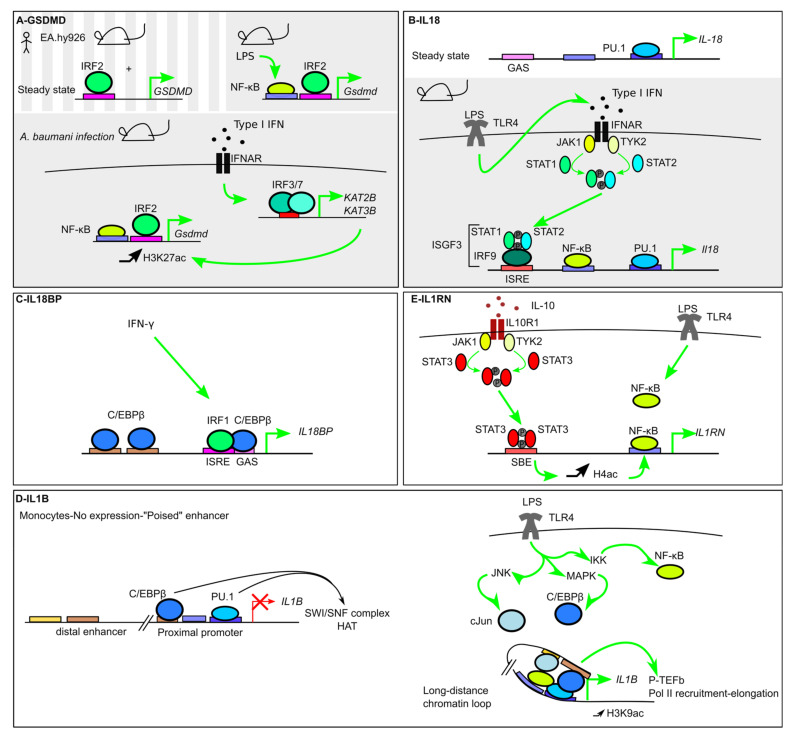
Transcriptional regulation of inflammasome substrates. (**A**) In mice and in the human endothelial EA.hy926 cell line, *GSDMD* constitutive expression is controlled by IRF2. During infection, NF-κB increases *Gsdmd* expression. Furthermore, induction of the histone acetyl transferases KAT2B and KAT3B, in an Interferon-α/β Receptor-(IFNAR)/IRF3/7-dependent manner, correlates with an increase in H3K27ac epigenetic mark at the *Gsdmd* promoter and an increase in its transcription level. (**B**) *IL18* is expressed at steady state and its promoter demonstrates PU.1 binding. LPS treatment leads to activation of STAT1 and STAT2 downstream of IFNAR, to the formation of the IFN-stimulated gene factor 3 (ISGF3) complex, containing IRF9, Phospho-STAT1, and Phospho-STAT2, and its binding to the *Il18* promoter. (**C**) *IL18BP* is induced by IFN-γ. IRF1 binds C/EBPβ to promote its recruitment to a Gamma-activated site (GAS) site. (**D**) *IL1b* is not expressed at steady state although PU.1 and C/EBPβ cooperate in monocytes to recruit the Switch/sucrose nonfermenting (SWI/SNF) complex and Histone acetyl transferase (HAT) to render the enhancer in a “poised” state. LPS treatment triggers C/EBPβ and c-Jun recruitment at the distal enhancer and the formation of a long-distance loop allowing the TFs to cooperate to increase H3K9ac, recruit positive transcription elongation factor b (p-TEFb) and activate Pol II. (**E**) IL-10 induces *IL1RN* expression in a STAT3-dependent manner. STAT3 binding to STAT-Binding Element (SBE) increases Histone H4 acetylation and promotes NF-κB binding.

As a core component of inflammasomes, *GSDMD* is constitutively expressed in numerous tissues. *Irf2* was identified in a forward genetic screen with a chemical mutagen, N-ethyl-N-nitrosourea (ENU), as a gene required for IL-1β release in response to Pam3CSK4 and ATP stimulation [23]. Similarly to the regulation of *CASP4* described above, IRF2 regulates the expression of *GSDMD* at steady state [23]. IRF2 binds an ISRE sequence in the *GSDMD* promoter immediately upstream of the TSS. *Irf2*^−/−^ mice do not express *Gsdmd*, and *Irf2*^−/−^ BMDMs display a profound inflammasome defect. In human cells, *IRF2* knockout in EA.hy926 cells confirmed the key role of IRF2 in controlling *GSDMD* expression [23]. We did not observe a major impact in the monocytic U937 cell line in the absence of IRF2 [22], suggesting that IRF2 regulates *GSDMD* in a cell-type-specific manner [88]. IRF1 and IRF2 have the same binding specificity for the ISRE sequence. Accordingly, and as described above for *CASP4* regulation, in the absence of IRF2, IRF1 plays a compensatory role and partially controls *GSDMD* expression (Figure 4A) [23].

*GSDMD* expression is largely constitutive. Yet, infections or pro-inflammatory signals increase *GSDMD* expression. Indeed, *Acinetobacter baumannii* infection induces a 2–4-fold increase in *Gsdmd* transcript in the liver and in BMDMs in an IFNAR1- and IRF3/7-dependent manner [106]. This induction correlates with the type I IFN-mediated induction of two histone lysine (K)-acetyltransferases, KAT2B and KAT3B/P300, and with an increase in the epigenetic histone modification H3K27ac at the *Gsdmd* promoter [106]. In addition, NF-κB activation following LPS stimulation promotes a 2-fold induction of *Gsdmd* transcript in murine adipocytes. Two NF-κB binding sites are present in the proximal *Gsdmd* promoter, and RelA/p65 binding contributes to *Gsdmd* induction [107].

Melatonin, a hormone maintaining the circadian rhythm, blunts LPS-mediated NF-κB activation and downregulates the expression of several inflammasome genes including *Gsdmd* in vitro and in vivo [107]. Together with the role of NR1D1 in the regulation of *Nlrp3* developed above, this regulation emphasizes the role of circadian rhythm in inflammasome transcriptional regulations.

### 7.2. IL-18 and IL-18BP

*IL18* expression is largely constitutive in humans and in mice [108]. *Il18* promoter includes a PU-box (a purine-rich sequence binding PU.1), NF-κB-recognition sequences, an ISRE site that was originally described to promote IRF-8-dependent induction of *IL18* in macrophages [109] and GAS elements [110].

Accordingly, *IL18* expression in human monocytes is inducible by LPS stimulation in a JAK/STAT-dependent manner. *IL18* induction requires both NF-κB activation and type I IFN signaling, resulting in delayed *IL18* induction kinetics compared to the kinetics of *IL1b* induction (an immediate early gene) [110]. Similarly, type I IFN signaling is critical for *Il18* induction in murine BMDMs treated with LPS. STAT1 and IRF9 are specifically required for this activity. The involvement of STAT1 and IRF9 suggests that IFN-stimulated gene factor 3 (ISGF3), a ternary protein complex made of IRF9, STAT1, and STAT2 and functioning as a transcription factor downstream of type I IFN receptors, is a direct regulator of *Il18* expression (Figure 4B) [111].

In murine epithelial cells, *Il18* homeostatic expression is controlled by IL-22. IL-22 is a cytokine from the IL-10 family produced by T cells and innate lymphoid cells (ILC) that induces antimicrobial and tissue-protective responses in epithelia. Interestingly, IL-22 regulates homeostatic expression of *Il18* in an organ-specific manner since *Il22*- and *IL22R1*-deficient mice display only a deficient *Il18* mRNA expression in the ileum but not in the colon, spleen, and lung. IL-22 also increases *Il18* expression during *T. gondii* infection [112]. Finally, *Il18* expression in the colon and the liver is impacted by the microbiota. Indeed, intestinal dysbiosis (i.e., gut microbiome imbalance) triggers upregulation of *Il18* in the liver. Conversely, germ-free mice have a down-regulation of *Il18* mRNA levels in the colon [113].

IL-18 bioavailability is regulated by IL-18BP (IL-18 binding protein), a decoy receptor, whose expression is strongly inducible by IFN-γ. IL-18BP binds IL-18 limiting the free IL-18 able to bind IL-18R to trigger signaling. *IL18BP* induction corresponds to a negative feedback loop since IL-18 is a major activator of IFN-γ production. *IL18BP* promoter includes a GAS, an ISRE and two C/EBPβ sites [114]. IRF-1 was identified as the main IRF responsible for *IL18BP* induction downstream of IFN-γ signaling [114]. IRF-1 forms a complex with C/EBPβ and directs its binding to a GAS site (Figure 4C). Interestingly, STAT1 is directly involved in *IL18BP* regulation in specific cell types [115]. *IL18BP* induction in response to IFN-γ is higher in epithelial cells than in monocytes. This differential induction is linked to differential methylation of a CpG island in the *IL18BP* promoter resulting in variable histone H3K9 acetylation and Pol II recruitment. Epigenetic modifications thus control cell-specific *IL18BP* production, likely antagonizing the IL-18/IFN-γ axis with different kinetics at systemic and epithelial barrier sites [115].

### 7.3. IL-1b and IL-1RA

*IL18* and *IL1b* display different regulations. Indeed, *IL1b* is not expressed at steady state but is strongly inducible following microbial (e.g., LPS) or sterile (e.g., TNF) pro-inflammatory signals.

*IL1b* transcription is regulated by three main regions: a proximal promoter, a distal one, and an enhancer site located ≈3 kb upstream of the TSS. Lineage-specific TFs control the cell-specific *IL1b* transcription and cooperate with signal-dependent TFs to trigger fast responses to pro-inflammatory signals. *IL1b* expression is most prominent in monocytes/macrophages, we will thus first present the regulation in these cells before presenting the atypical regulation that can occur in other cell types.

#### 7.3.1. Roles of the Lineage-Specific/Pioneer TFs PU.1 and C/EBPβ

*IL1B* is primarily expressed in monocytes and macrophages [116]. The binding of the hematopoietic-specific transcription factor PU.1 on several PU-boxes in the proximal *IL1B* promoter and in the enhancer is largely responsible for the cell-specific expression of *IL1B*. Indeed, ectopic expression of PU.1 in HeLa cells is sufficient to drive *IL1B* expression. PU.1 is a pioneer TF, which recruits the SWI/SNF family chromatin remodeling complex [117]. Similarly, C/EBPβ initiates chromatin opening through its ability to bind the histone acetyl transferase p300/CBP and the SWI/SNF complex (Figure 4D) [118]. Like PU.1, C/EBPβ is predominantly expressed in monocytes/macrophages and its binding in the proximal promoter is required for *IL1B* expression [116]. PU.1 and C/EBPβ act in collaboration to generate a highly accessible *IL1B* promoter (without nucleosome blocking the access of Pol II), yet without Pol II binding at steady state [119]. This “poised” chromatin structure is likely responsible for the ability of *IL1B* promoter to be rapidly turned-on following pro-inflammatory signals [120]. c-Jun also binds constitutively on AP-1 (activator protein 1) sites in the proximal enhancer and likely acts in concert with PU.1 and C/EBPβ to remodel chromatin at this site [120]. *IL1B* is an immediate early gene meaning that its expression is upregulated immediately post pro-inflammatory signals independent of protein neosynthesis.

#### 7.3.2. Signal-Dependent TFs (NF-κB)

These pioneer TFs act in synergy with multiple other TFs that are either bound constitutively or in a signal-dependent manner to the enhancer, the promoter, or the intronic sequences of *IL1b*. In addition to PU.1, C/EBPβ, and cJun, NF-κB, AP-1 (cJun/cFos heterodimer), STAT proteins, and IRF4/8 bind *IL1B* regulatory regions [21,120,121,122]. IRF4 and IRF8 participate in the dynamic transcription of *IL1B* following LPS treatment in human monocytes [120]. The relevance of these two TFs remains unclear since *Irf8*-deficient macrophages do not present any obvious *Il1b* defect [25] and since ectopic expression of *Irf4* or *Irf8* in murine macrophages inhibits *Il1b* expression [21].

The role of NF-κB is clearly established following treatments with microbial (e.g., LPS) or sterile pro-inflammatory stimuli (e.g., IL-1β in a feedforward loop). *Il1b* induction downstream of TLRs is largely mediated by MYD88, although a moderate TRIF-dependent induction is observed following TLR3 engagement by poly(I:C) [111]. p65/RelA is recruited to the *IL1B* promoter in a signal-dependent manner, while c-Jun, which expression is induced by MAPK downstream of TLRs, is both constitutively bound to the proximal *IL1B* enhancer and inducibly recruited to both the distal enhancer and the promoter [120].

The inducible expression of *IL1B* in response to pro-inflammatory signals is linked to an enhancer region located -3757 and -2729 bp upstream of the TSS. The induction is dependent on the binding of C/EBPβ at this enhancer site [123] and on a long-range chromatin looping that allows C/EBPβ to directly interact with PU.1, which is bound to the proximal promoter [124]. In addition, binding of NF-κB subunits (RelA/p65, NF-κB1 (p50), c-Rel (p85)) in the proximal promoter region contributes to *IL1B* induction [125]. NF-κB interacts with PU.1 [124]. LPS-induced *IL1B* transcription thus results from the intimate cooperation of the signal-dependent TF NF-κB with the pioneer TFs C/EBPβ and PU.1. C/EBPβ recruits the positive transcriptional elongation factor P-TEFb at the *IL1B* promoter. P-TEFb phosphorylates Pol II C-terminal tail and promotes transcription elongation [124].

*IL1B* promoter also includes a composite cAMP response element (CRE)/C/EBPβ site [126] located between -2755 and -2762. The CRE binds CREB and the related TF ATF-1 following LPS addition [127]. Increase in intracellular cAMP results in the activation of PKA and in the subsequent phosphorylation of CREB, which interacts with C/EBPβ [128] to induce *IL1B* expression. In agreement, Prostaglandin E2 (PGE2), by signaling through the Prostaglandin E2 receptors 2 and 4 (EP2 and EP4), induces an increase in intracellular cAMP and triggers *IL1B* induction [129,130]. Since IL-1β induces PGE2 production through the transcriptional control of *COX2* (the inducible enzyme producing PGE2). This regulation illustrates another feedforward loop that could be key in mounting potent inflammatory responses [130].

#### 7.3.3. Metabolic Regulation of IL1b

In addition to the direct NF-κB-mediated effect, LPS stimulation triggers *IL1b* induction through metabolic rewiring. Indeed, LPS treatment is associated with a macrophage metabolism shift from mitochondrial oxidative phosphorylation to aerobic glycolysis. This shift is associated with a strong increase in the intracellular concentration of succinate, an intermediate metabolite of the tricarboxylic acid (TCA) cycle. Succinate stabilizes hypoxia-inducible factor 1α (HIF1α) protein. Following LPS stimulation, HIF1α binds to the hypoxia responsive element (HRE) located ≈300 pb upstream of the *Il1b* TSS. Intracellular succinate thus acts as an endogenous danger signal connecting metabolic rewiring following chronic LPS stimulation and *Il1b* transcription (together with other inflammatory genes) [131]. HIF1α appears as a hub integrating numerous signals to induce *Il1b*. Indeed, in addition to sensing intracellular succinate concentration, HIF1α function requires its upregulation by NF-κB or by LXRα in human macrophages. LXR are nuclear receptors sensing cholesterol derivatives and activated in atherosclerotic plaques. Addition of an LXR agonist increases LXR binding at the HRE sites located in the *IL1B* and the *HIF1α* promoters, suggesting that an LXR/HIF1α complex binds at these sites [132]. In addition, hypoxia also increases LPS-induced IL-1β release, in agreement with *IL1B* being a direct target of HIF1α.

NRF2 (NF-E2-related factor-2) is a transcription factor activated by oxidative stress. At steady state, NRF2 is degraded due to its binding to KEAP1, a E3 ubiquitin ligase adaptor. During oxidative stress, KEAP1 dissociates from NRF2 allowing NRF2 to accumulate and translocate into the nucleus. In the nucleus, NRF2 predominantly binds ARE (antioxidant responsive element) to positively regulate transcription. NRF2 binds DNA upstream of *Il1b* TSS, in the enhancer region. Surprisingly, NRF2 binding is independent of ARE sites, suggesting that NRF2 might be recruited via interaction with another TF. Furthermore, NRF2 activation correlates with a decrease in Pol II recruitment at the *Il1b* TSS [133]. NRF2 may thus have an atypical activity (ARE-independent repression) at the *Il1b* promoter to decrease inflammation in conditions of high oxidative stress.

#### 7.3.4. Negative Regulation of IL1b

*Il1b* expression is negatively regulated by IL-10 in a STAT3-dependent manner. Type I IFNs, which induce IL-10, also negatively regulate *IL1b* transcription in murine BMDMs and in human primary monocytes [26].

IL-19, -20, and -24 are cytokines belonging to the IL-10 family, produced by myeloid and epithelial cells and signaling through the type I IL-20 receptor (IL-20R). These cytokines negatively regulate *Il1b* transcription in murine keratinocytes exposed to *Staphylococcus aureus*. IL20R signaling leads to a decrease in STAT3 and in C/EBPβ active isoforms. Meanwhile, C/EBPβ displays increasing inhibitory sumoylation. The shift in the balance between C/EBPβ active and inactive isoforms correlates with a decrease in C/EBPβ binding at the *Il1b* promoter [134].

#### 7.3.5. IL1b Regulation in T Cells

Several studies [135,136,137] have demonstrated a production of IL-1β in CD4^+^ T cells using elegant cell-type-specific gene ablation and bone-marrow transfer experiments [136,137]. IL-1β produced by T cells contributes to chronic inflammatory diseases such as experimental autoimmune encephalomyelitis [136]. *IL1B* expression in CD4^+^ T cells (and particularly the CCR5^+^ subset) is upregulated following TCR engagement with different costimulation signals. CD4^+^ T cells do not present detectable amounts of PU.1 protein [138] even after TCR engagement. Accordingly, *IL1B* promoter in resting T cells is not accessible and does not present the poised architecture observed in monocytes [119]. In agreement with this difference, *Il1b* expression is much lower in activated T cells than in activated monocytes (≈1000-fold at the transcript level). The mechanism leading to TCR-mediated and PU.1-independent induction of *Il1b* in CD4^+^ T cells remains unclear. This mechanism is associated with an increase in the activating epigenetic histone modifications (H3K4me3 and H3K9ac), a corresponding decrease in the inhibiting modification (H3K27me3) at the *Il1b* promoter site and Pol II enrichment throughout *Il1b* gene upon T cell activation. In contrast to housekeeping genes transcribed at high levels, inhibitory H3K27me3 marks persist on the *Il1b* promoter resulting in a bivalent H3K4me3^+^/H3K27me3^+^ low-activity promoter [138].

#### 7.3.6. IL1b Regulation in DCs

As discussed above, conventional DCs are largely deficient in inflammasome responses due to IRF4/IRF8-mediated negative regulation of inflammasome genes transcription [21,51]. IL-21 is a cytokine produced by follicular helper (Tfh), Th17, and NK cells that triggers STAT3 activation. In cDCs, but not in BMDMs, IL-21 (or IL-10) increases *Il1b* transcription in a *Stat3*-dependent manner. Although *Il1b* induction (≈3-fold) remains modest compared to *Il1b* induction induced by NF-κB activation in BMDMs, IL-21 contributes to *Il1b* expression in vivo during Pneumonia Virus of Mice (PVM) infection, indicating that this production is likely relevant in specific contexts [139]. This IL-21/IL-10-Stat3-dependent increase in *Il1b* expression illustrates how one cytokine can have a differential impact in two different cell types (cDCs, BMDMs).

Overall, *IL1B* enhancers and promoters integrate signals from multiple cytokines either directly (e.g., IL-1β autoamplification loop) or indirectly (type I IFN inducing IL-10), from intrinsic (e.g., circadian clock), environmental (xenobiotics), metabolic (e.g., succinate) or infectious (LPS) signals. *IL1B* regulation has been mostly studied in monocytes/macrophages, in which *IL1B* is an immediate gene strongly upregulated following pro-inflammatory signals detection. Yet, there is now clear evidence that low expression of *IL1b* in DCs or CD4^+^ T cells, although controlled by different transcriptional mechanisms, is relevant in chronic inflammatory diseases [140].

#### 7.3.7. IL1RN

*IL1RN* encodes IL-1 receptor antagonist, a secreted cytokine that competes with IL-1α and IL-1β for their binding to the IL-1 receptor. In contrast to IL1α/β, IL1Ra does not trigger IL-1 receptor signaling. The expression level of *IL1RN* thus balances IL-1 bioactivity [141].

*IL1RN* expression is inducible by LPS [142], IL-1α/β (acting as a negative feedback loop) [143] and IFN treatment [144]. Like *IL1B*, *IL1RN* expression is controlled by C/EBPα/β, PU.1, and NF-κB that bind to the *IL1RN* promoter [143]. IL-10 also induces *IL1RN* expression in a STAT3-dependent manner to dampen inflammation. IL-10 triggers recruitment of STAT3 to the *IL1RN* promoter and synergizes with LPS to promote the recruitment of NF-κB p50/p65 and Pol II. The increased NF-κB recruitment following IL-10 addition correlates with histone H4 acetylation suggesting that STAT3 recruitment at the *IL1RN* promoter modifies chromatin structure and accessibility to NF-κB binding sites [145] (Figure 4E).

## 8. Conclusions

**Figure 5:**
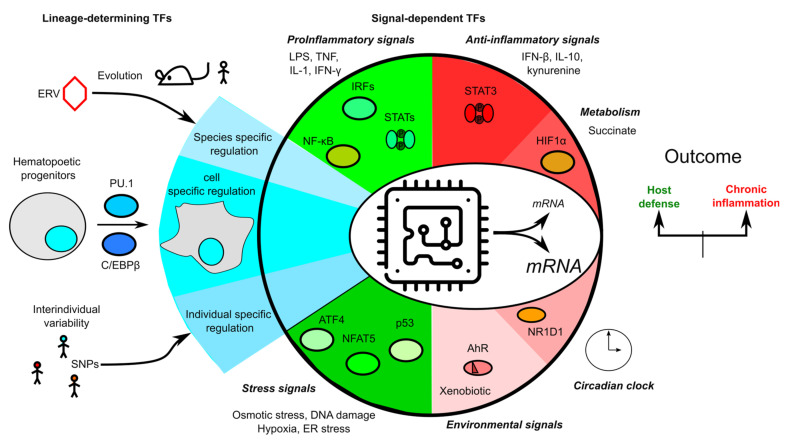
Overview of the transcriptional regulation of inflammasome genes. Transcription is schematized as a microchip integrating multiple signals from evolution (e.g., endogenous retroviruses), lineage engagement and differentiation, individual single nucleotide polymorphisms (SNPs), the environment, metabolism, stress responses, and pro- and anti-inflammatory immune signaling pathways to control inflammasome transcripts levels.

While there is no doubt that post-transcriptional regulations of inflammasomes are intimately linked to their activation, these regulations take place in a transcriptional landscape (Table 1) that shapes the output responses (Figure 5). At steady state, the transcriptional regulation of inflammasome genes is the result of a complex cascade of events taking place during lineage differentiation in a species-specific manner. As observed in cDCs, these regulations have a profound impact on the biological responses by limiting pyroptosis and favoring T cell priming and adaptive immune responses [21]. Furthermore, in inflammatory conditions, the upregulation of inflammasome genes (as exemplified by *Casp11* and the sensitivity to LPS [99]) strongly modifies the ability of the host to detect and respond to PAMPs. Importantly, signal-induced transcriptional regulations can be partly fixed in time due to epigenetic reprogramming/immunological imprinting. These long-term changes are responsible for the trained immunity (also called innate immune memory). They affect inflammasome genes [146] and contribute to the protective effect of trained immunity against pathogens [147] or possibly to chronic inflammation [148].

Although specific inflammasome genes have specific regulations, there are also common regulations that globally affect the inflammasome responses either positively (e.g., NF-κB activation) or negatively (IRF4/8 in cDCs). These specific inflammasome gene regulations likely shape the biological inflammasome response (balance between IL-1β and IL-18, balance between cytokine release and pyroptosis, and balance between apoptosis and pyroptosis). Similarly, the clinical phenotypes resulting from gain of function mutations in inflammasome genes differ based on the mutated sensor. The prevalence of organ-specific phenotypes (e.g., cutaneous phenotypes in NLRP1-associated syndromes [149,150]) or cytokine-driven diseases (IL-18 in NLRC4-associated syndromes [151] compared to IL-1 in NLRP3/Cryopyrin-Associated periodic Syndromes [152]) may be largely due to differential transcriptional regulations. In addition to monogenic diseases, the fine understanding of the networks of DNA motifs, chromatin structure, and transcription factors that regulate inflammasome genes may help us understanding how single nucleotide polymorphisms (SNPs) could impact inflammasome regulation [153] and predispose human beings to various inflammatory diseases. Selective inhibitors of histone modifiers have been developed [154] with an impact on the pro-inflammatory response of human macrophages. Developing specific therapeutic intervention targeting transcription regulation is a fascinating challenge for the field.

## Figures and Tables

**Table 1:** Main Transcription Factors (TFs) Regulating Inflammasome Genes.

TF	Context	Target	Species	Outcome
AhR	Xenobiotics, metabolites	*Nlrp3*	m	Anti-inflammatory
ATF4	ER stress	*NLRP1*	h	Pro-inflammatory
BLIMP1	Human steady state	*AIM2*	h	Negative regulation
C/EBPβ	Cell differentiation	*MEFV, IL1B, IL1RN*	h	Cell-specific expression
CHOP	ER stress	*Casp11*	m	LPS-induced lung inflammation
CREB	PGE2 signaling	*IL1B*	h	Pro-inflammatory
GFI1	Negative feedback loop	*Nlrp3*	m	Anti-inflammatory
HIF1α	Metabolism/Hypoxia	*IL1B*	h, m	Pro-inflammatory
IRF1	IFN-Υ treatment	*CASP1*	h, m	Pro-inflammatory
IRF1	IFN-Υ treatment	*IL18BP*	h	Anti-inflammatory
IRF1/2	IFN-Υ treatment	*AIM2*	h	Pro-inflammatory
IRF2	Steady state	*CASP4*	h	Inflammasome competence
IRF2	Steady state	*GSDMD*	h, m	Inflammasome competence
IRF4	cDC1-steady state	*Nlrc4, Il1b, Pycard*	m	Antigen presentation
IRF8	cDC2-steady state	*Nlrp3, Nlrc4, Pycard, Il1b*	m	Antigen presentation
IRF8	BMDM-steady state	*Naip2, 5, 6, Nlrc4*	m	Resistance to *Salmonella*
IRF8	EBV + lymphoblastoid cells	*CASP1*	h	EBV lytic cycle
ISGF3	type I IFN response	*Il18*	m	LPS-mediated induction
LXRα	Metabolism	*IL1B*	h	Pro-inflammatory
NF-κB	Pro-inflammatory signals	*NLRP3, MEFV, CASP4, CASP5, Casp11, Gsdmd, IL18, IL1B, IL1RN*	h, m	Kinetics of inflammasome response
NFAT5	Osmotic stress	*Nlrp3*	m	Pro-inflammatory
NR1D1	Circadian clock	*Nlrp3*	m	Circadian oscillation
NRF2	Oxidative stress	*Il1b*	m	Pro-inflammatory
p53	DNA damage	*Casp11, CASP1, NLRC4*	m, h	Pro-inflammatory
PU.1	Cell differentiation	*IL-18, IL1B, IL1RN*	h	Cell-specific expression
SREBP-1A	NF-κB activation	*Nlrp1a*	m	Metabolic inflammation
STAT1	IFN-Υ treatment	*AIM2*	h	Induction
STAT1	IFN-Υ treatment	*IL18BP*	h	Anti-inflammatory
STAT3	IL-10, IL-20R family	*Il1b*	m	Anti-inflammatory
STAT3	IL-21 in DC	*Il1b*	m	Resistance to pneumonia Virus of mice

TFs highlighted in gray have a negative effect. See text for details and references. “m” stands for mouse, “h” for human.

## Abbreviations

AhR	Aryl hydrocarbon receptor
AIM2	Absent in melanoma 2
AP-1	Activator protein-1
ARE	Antioxidant responsive element
ASC	Apoptosis-associated speck-like protein containing a card
ATF	Activating transcription factor
BLIMP1	B lymphocyte-induced maturation protein-1
BMDM	Bone marrow-derived macrophage
BCL6	B cell lymphoma 6
Brd4	Bromodomain-containing protein 4
CHOP	C/ebp homologous protein
CRE	Camp-response element
CREB	Camp response element-binding protein
DC	Dendritic cell
DSS	Dextran sodium sulfate
EBV	Epstein–Barr virus
EICE	Ets-irf composite element
ENU	N-ethyl-n-nitrosourea
ERV	Endogenous retrovirus
ETS	Erythroblast transformation specific
GAS	Gamma-activated site
GFI1	Growth factor independence 1
GRE1	Gli-responsive element
HAMPs	Homeostasis-altering molecular processes
HAT	Histone acetyl transferase
HIF1α	Hypoxia-inducible factor 1α
HRE	Hypoxia response element
IFNAR	Interferon-α/β receptor
IRE-1	Inositol-requiring enzyme 1
IRF	Interferon regulatory factor
ISRE	Ifn-stimulated response element
LTR	Long terminal repeat
NAIP	Neuronal apoptosis inhibitory protein
NFAT5	Nuclear factor of activated t cells 5
NF-κB	Nuclear factor κb
NLR	Nucleotide-binding domain and leucine-rich repeat containing
NLRP	Nlr family pyrin domain containing
Nod2	Nucleotide-binding oligomerization domain 2
NR1D1	Nuclear receptor subfamily 1 group d member 1
Nrf2	Nf-e2-related factor 2
ORE	Osmotic response element
PAMP	Pathogen-associated molecular pattern
PARP1	Poly [ADP-ribose] polymerase 1
PERK	Pkr-like er protein kinase
P-TEFb SBE	Positive transcription elongation factor b STAT-Binding Element
SREBP-1a	Sterol regulatory element binding protein-1a
STAT1	Signal Transducer and Activator of Transcription 1
SWI/SNF	Switch/sucrose nonfermenting
TCA	Tricarboxylic acid
TF	Transcription factor
TLR	Toll-like receptor
TNF	Tumor necrosis factor
TSS	Transcription start site
UPR	Unfolded protein response
XRE	Xenobiotic response elements
